# Diaqua­bis­(4,4′-bipyridine-κ*N*)bis­(2,4,5-trifluoro-3-hy­droxy­benzoato-κ*O* ^1^)manganese(II)

**DOI:** 10.1107/S1600536811049610

**Published:** 2011-11-25

**Authors:** Chun-Gang Min, Xi-Kun Yang

**Affiliations:** a Research Center for Analysis and Measurement, Kunming University of Science and Technology, 650093 Kunming, Yunnan, People’s Republic of China

## Abstract

In the title compound, [Mn(C_7_H_2_F_3_O_3_)_2_(C_10_H_8_N_2_)_2_(H_2_O)_2_], the Mn^II^ ion, situated on a centre of inversion, has a distorted octa­hedral coordination geometry and is coordinated by two N atoms from two 4,4′-bipyridine ligands, two O atoms from two 2,4,5-trifluoro-3-hy­droxy­benzoate ligands and two water mol­ecules. Inter­molecular O—H⋯N hydrogen bonds link the mol­ecules into a chain along the *a* axis. Inter­actions between neighboring chains occur through O—H⋯O hydrogen bonds, which link the chains into a two-dimensional supra­molecular network parallel to the * ac* plane. In addition, O—H⋯O hydrogen bonds between the water mol­ecules and carboxyl­ate groups also exist in the the crystal structure.

## Related literature

For general background to the design and synthesis of novel metal-organic coordination polymers based on fluoro­benzoic acid, see: Gielen *et al.* (1992 ▶); Ma *et al.* (2006 ▶); Shi *et al.* (2011 ▶). For a related structure, see: Zhu (2009 ▶).
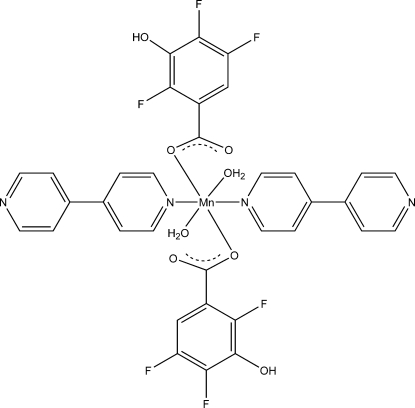


## Experimental

### 

#### Crystal data

[Mn(C_7_H_2_F_3_O_3_)_2_(C_10_H_8_N_2_)_2_(H_2_O)_2_]
*M* *_r_* = 785.51
Triclinic, 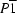

*a* = 7.0706 (6) Å
*b* = 8.2939 (7) Å
*c* = 13.9856 (12) Å
α = 79.200 (1)°
β = 88.338 (1)°
γ = 79.830 (2)°
*V* = 792.96 (12) Å^3^
*Z* = 1
Mo *K*α radiation
μ = 0.51 mm^−1^
*T* = 298 K
0.30 × 0.25 × 0.20 mm

#### Data collection

Bruker SMART APEXII CCD area-detector diffractometer
Absorption correction: multi-scan (*SADABS*; Bruker, 2005 ▶) *T*_min_ = 0.861, *T*_max_ = 0.904
4185 measured reflections
2792 independent reflections
2101 reflections with *I* > 2σ(*I*)
*R*_int_ = 0.041

#### Refinement

*R*[*F* ^2^ > 2σ(*F* ^2^)] = 0.038
*wR*(*F* ^2^) = 0.084
*S* = 1.01
2792 reflections
241 parameters
H-atom parameters constrained
Δρ_max_ = 0.29 e Å^−3^
Δρ_min_ = −0.28 e Å^−3^

### 

Data collection: *APEX2* (Bruker, 2005 ▶); cell refinement: *SAINT* (Bruker, 2005 ▶); data reduction: *SAINT*; program(s) used to solve structure: *SHELXTL* (Sheldrick, 2008 ▶); program(s) used to refine structure: *SHELXTL*; molecular graphics: *SHELXTL*; software used to prepare material for publication: *SHELXTL*.

## Supplementary Material

Crystal structure: contains datablock(s) global, I. DOI: 10.1107/S1600536811049610/rn2092sup1.cif

Structure factors: contains datablock(s) I. DOI: 10.1107/S1600536811049610/rn2092Isup2.hkl

Additional supplementary materials:  crystallographic information; 3D view; checkCIF report

## Figures and Tables

**Table 1:** Hydrogen-bond geometry (Å, °)

*D*—H⋯*A*	*D*—H	H⋯*A*	*D*⋯*A*	*D*—H⋯*A*
O1*W*—H2*W*⋯O2	0.85	2.04	2.839 (2)	155
O1*W*—H1*W*⋯O2^i^	0.84	1.97	2.773 (2)	159
O3—H3⋯N2^ii^	0.82	1.89	2.641 (3)	152

Symmetry codes: (i) 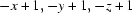; (ii) 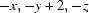.

**Fig. 1.**
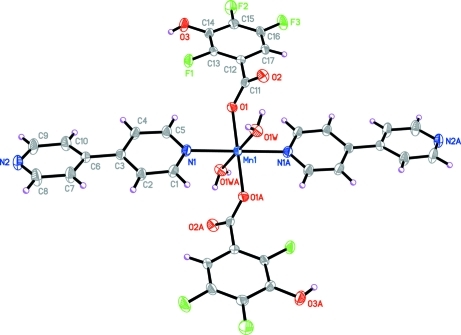
The molecular structure of (I), with displacement ellipsoids drawn at the 30% probability level [symmetry codes: (A) -x, 1 - y, 1 - z].

**Fig. 2.**
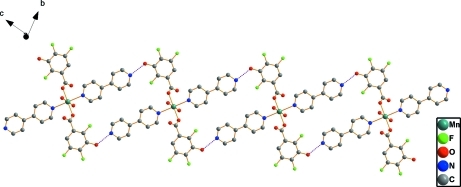
The linear chain of (I). All H atoms have been omitted for clarity

**Fig. 3.**
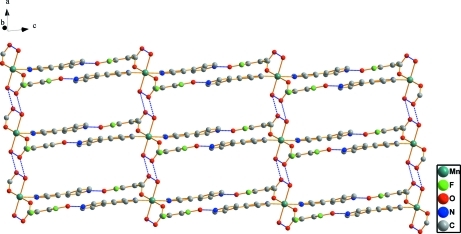
The two-dimensional of (I). All H atoms have been omitted for clarity

## supplementary crystallographic information

### Comment

In recent years, the design and synthesis of novel metal-organic coordination polymers based on fluorobenzoic acids have attracted much attention (Gielen *et al.*, 1992; Ma *et al.*, 2006; Zhu, 2009; Shi *et al.*, 2011). We report herein the crystal structure (Fig. 1) of the title compound (I) based on 2,4,5-trifluoro-3-hydroxy-benzoic and 4,4'-bipyridine. In the crystal packing, the adjacent mononuclear units are linked into a linear chain along *a* axis *via* O—H···N hydrogen bonds (Fig. 2). Furthermore, additional interactions within neighboring chains occur through O—H···O hydrogen bonds, thus a two-dimensional supramolecular network parallel to *ac* plane is formed, as shown in Fig. 3. In addition, intramolecular O—H···O hydrogen bonds (O1W—H2W···O2) between the water molecules and carboxylate groups also exist in the the crystal structure.

### Experimental

A mixture of Mn(CH_3_COO)_2_^.^4H_2_O (0.1 mmol), 2,4,5-trifluoro-3-hydroxy-benzoic acid (0.2 mmol), Et_3_N (0.1 ml), EtOH (3 ml) and H_2_O (2 ml) was sealed in a 10 ml Teflon-lined stainless-steel reactor, heated to 393 K for 72 h, and then slowly cooled to room temperature. Light yellow block crystals suitable for X-ray diffraction analysis were collected by filtration.

### Refinement

H atoms attached to C atoms were placed in calculated positions (C—H = 0.93 Å) and refined as riding atoms and with *U*_iso_(H) = 1.2 *U*_eq_(C),respectively. The hydroxyl and water H atoms were located in a difference map and refined with O—H bond length from 0.82 to 0.85 Å and *U*_iso_(H) = 1.5 *U*_eq_(O).

### Crystal data

**Table:**

[Mn(C_7_H_2_F_3_O_3_)_2_(C_10_H_8_N_2_)_2_(H_2_O)_2_]	*Z* = 1
*M**_r_* = 785.51	*F*(000) = 399
Triclinic, *P*1	*D*_x_ = 1.645 Mg m^−^^3^
Hall symbol: -P 1	Mo *K*α radiation, λ = 0.71073 Å
*a* = 7.0706 (6) Å	Cell parameters from 1483 reflections
*b* = 8.2939 (7) Å	θ = 2.9–26.6°
*c* = 13.9856 (12) Å	µ = 0.51 mm^−^^1^
α = 79.200 (1)°	*T* = 298 K
β = 88.338 (1)°	Block, light yellow
γ = 79.830 (2)°	0.30 × 0.25 × 0.20 mm
*V* = 792.96 (12) Å^3^	

### Data collection

**Table:**

Bruker SMART APEXII CCD area-detector diffractometer	2792 independent reflections
Radiation source: fine-focus sealed tube	2101 reflections with *I* > 2σ(*I*)
graphite	*R*_int_ = 0.041
φ and ω scans	θ_max_ = 25.1°, θ_min_ = 2.5°
Absorption correction: multi-scan (*SADABS*; Bruker, 2005)	*h* = −8→8
*T*_min_ = 0.861, *T*_max_ = 0.904	*k* = −8→9
4185 measured reflections	*l* = −15→16

### Refinement

**Table:**

Refinement on *F*^2^	Primary atom site location: structure-invariant direct methods
Least-squares matrix: full	Secondary atom site location: difference Fourier map
*R*[*F*^2^ > 2σ(*F*^2^)] = 0.038	Hydrogen site location: inferred from neighbouring sites
*wR*(*F*^2^) = 0.084	H-atom parameters constrained
*S* = 1.01	*w* = 1/[σ^2^(*F*_o_^2^) + (0.0281*P*)^2^ + 0.0001*P*] where *P* = (*F*_o_^2^ + 2*F*_c_^2^)/3
2792 reflections	(Δ/σ)_max_ < 0.001
241 parameters	Δρ_max_ = 0.29 e Å^−^^3^
0 restraints	Δρ_min_ = −0.28 e Å^−^^3^

### Special details

**Table:**

Geometry. All e.s.d.'s (except the e.s.d. in the dihedral angle between two l.s. planes) are estimated using the full covariance matrix. The cell e.s.d.'s are taken into account individually in the estimation of e.s.d.'s in distances, angles and torsion angles; correlations between e.s.d.'s in cell parameters are only used when they are defined by crystal symmetry. An approximate (isotropic) treatment of cell e.s.d.'s is used for estimating e.s.d.'s involving l.s. planes.
Refinement. Refinement of *F*^2^ against ALL reflections. The weighted *R*-factor *wR* and goodness of fit *S* are based on *F*^2^, conventional *R*-factors *R* are based on *F*, with *F* set to zero for negative *F*^2^. The threshold expression of *F*^2^ > σ(*F*^2^) is used only for calculating *R*-factors(gt) *etc*. and is not relevant to the choice of reflections for refinement. *R*-factors based on *F*^2^ are statistically about twice as large as those based on *F*, and *R*- factors based on ALL data will be even larger.

### Fractional atomic coordinates and isotropic or equivalent isotropic displacement parameters (Å^2^)

**Table:**

	*x*	*y*	*z*	*U*_iso_*/*U*_eq_	
Mn1	0.0000	0.5000	0.5000	0.03192 (17)	
F1	0.2679 (2)	0.40469 (16)	0.18986 (10)	0.0502 (4)	
F2	0.4119 (2)	−0.13268 (18)	0.11722 (11)	0.0654 (5)	
F3	0.4709 (2)	−0.26309 (17)	0.30543 (11)	0.0653 (5)	
O1	0.1454 (2)	0.3687 (2)	0.39423 (11)	0.0390 (4)	
O1W	0.2747 (2)	0.5272 (2)	0.55884 (12)	0.0481 (5)	
H1W	0.3355	0.6075	0.5531	0.072*	
H2W	0.3528	0.4491	0.5395	0.072*	
O2	0.4427 (2)	0.2766 (2)	0.45383 (12)	0.0452 (5)	
O3	0.3067 (3)	0.1957 (2)	0.05414 (12)	0.0541 (5)	
H3	0.2740	0.2973	0.0440	0.081*	
N1	−0.0323 (3)	0.7434 (2)	0.38644 (14)	0.0371 (5)	
N2	−0.2524 (3)	1.4900 (3)	0.03585 (15)	0.0475 (6)	
C9	−0.2397 (4)	1.3428 (3)	0.01136 (19)	0.0573 (8)	
H9	−0.2601	1.3389	−0.0535	0.069*	
C10	−0.1977 (4)	1.1936 (3)	0.07644 (19)	0.0513 (7)	
H10	−0.1919	1.0933	0.0549	0.062*	
C6	−0.1646 (3)	1.1932 (3)	0.17280 (17)	0.0340 (6)	
C7	−0.1801 (4)	1.3478 (3)	0.19819 (19)	0.0562 (8)	
H7	−0.1596	1.3557	0.2624	0.067*	
C8	−0.2257 (5)	1.4904 (3)	0.1290 (2)	0.0617 (9)	
H8	−0.2384	1.5927	0.1488	0.074*	
C3	−0.1171 (3)	1.0381 (3)	0.24554 (17)	0.0327 (6)	
C4	−0.0622 (4)	0.8826 (3)	0.22071 (18)	0.0458 (7)	
H4	−0.0530	0.8736	0.1554	0.055*	
C5	−0.0210 (4)	0.7414 (3)	0.29101 (18)	0.0465 (7)	
H5	0.0164	0.6394	0.2713	0.056*	
C1	−0.0822 (4)	0.8925 (3)	0.41036 (17)	0.0409 (6)	
H1	−0.0885	0.8980	0.4762	0.049*	
C2	−0.1251 (4)	1.0389 (3)	0.34485 (17)	0.0407 (6)	
H2	−0.1597	1.1390	0.3669	0.049*	
C11	0.3130 (4)	0.2840 (3)	0.39429 (16)	0.0322 (6)	
C12	0.3486 (3)	0.1745 (3)	0.31840 (16)	0.0305 (5)	
C13	0.3175 (3)	0.2371 (3)	0.22071 (17)	0.0331 (6)	
C15	0.3911 (4)	−0.0307 (3)	0.18248 (18)	0.0396 (6)	
C16	0.4213 (3)	−0.0955 (3)	0.27927 (18)	0.0395 (6)	
C17	0.4035 (3)	0.0031 (3)	0.34792 (17)	0.0355 (6)	
H17	0.4276	−0.0432	0.4132	0.043*	
C14	0.3377 (3)	0.1397 (3)	0.14978 (17)	0.0350 (6)	

### Atomic displacement parameters (Å^2^)

**Table:**

	*U*^11^	*U*^22^	*U*^33^	*U*^12^	*U*^13^	*U*^23^
Mn1	0.0402 (3)	0.0269 (3)	0.0264 (3)	−0.0019 (2)	−0.0003 (2)	−0.0029 (2)
F1	0.0817 (11)	0.0280 (8)	0.0353 (8)	−0.0021 (7)	0.0036 (8)	0.0012 (6)
F2	0.1059 (14)	0.0421 (10)	0.0487 (10)	0.0014 (9)	−0.0030 (9)	−0.0223 (8)
F3	0.0996 (13)	0.0275 (9)	0.0604 (11)	0.0124 (8)	−0.0129 (9)	−0.0065 (8)
O1	0.0435 (11)	0.0392 (10)	0.0310 (10)	0.0052 (8)	−0.0003 (8)	−0.0096 (8)
O1W	0.0412 (11)	0.0415 (11)	0.0618 (12)	−0.0062 (8)	−0.0004 (9)	−0.0109 (9)
O2	0.0460 (11)	0.0439 (11)	0.0478 (11)	−0.0067 (8)	−0.0111 (9)	−0.0128 (9)
O3	0.0902 (15)	0.0395 (11)	0.0288 (10)	−0.0025 (10)	−0.0028 (10)	−0.0042 (8)
N1	0.0477 (13)	0.0311 (12)	0.0310 (12)	−0.0070 (10)	0.0016 (10)	−0.0018 (9)
N2	0.0673 (16)	0.0354 (13)	0.0365 (13)	−0.0076 (11)	−0.0104 (11)	0.0021 (11)
C9	0.095 (2)	0.0457 (18)	0.0285 (15)	−0.0092 (16)	−0.0113 (15)	0.0005 (13)
C10	0.082 (2)	0.0330 (16)	0.0375 (16)	−0.0059 (14)	−0.0067 (14)	−0.0051 (13)
C6	0.0353 (14)	0.0322 (14)	0.0320 (14)	−0.0039 (11)	−0.0009 (11)	−0.0015 (11)
C7	0.095 (2)	0.0367 (17)	0.0334 (16)	−0.0049 (15)	−0.0119 (15)	−0.0031 (13)
C8	0.107 (3)	0.0311 (16)	0.0451 (18)	−0.0064 (16)	−0.0170 (17)	−0.0033 (14)
C3	0.0331 (14)	0.0309 (14)	0.0329 (14)	−0.0065 (11)	0.0002 (11)	−0.0024 (11)
C4	0.072 (2)	0.0352 (16)	0.0276 (14)	−0.0041 (13)	0.0043 (13)	−0.0038 (12)
C5	0.073 (2)	0.0285 (15)	0.0345 (16)	−0.0011 (13)	0.0019 (14)	−0.0046 (12)
C1	0.0605 (18)	0.0342 (15)	0.0283 (14)	−0.0101 (12)	0.0006 (12)	−0.0046 (12)
C2	0.0596 (17)	0.0270 (14)	0.0339 (15)	−0.0057 (12)	0.0003 (12)	−0.0038 (12)
C11	0.0408 (15)	0.0265 (13)	0.0274 (13)	−0.0059 (11)	0.0026 (12)	−0.0002 (10)
C12	0.0277 (13)	0.0318 (14)	0.0308 (13)	−0.0026 (10)	0.0028 (10)	−0.0061 (11)
C13	0.0363 (14)	0.0219 (13)	0.0371 (14)	−0.0003 (10)	0.0027 (11)	−0.0002 (11)
C15	0.0461 (16)	0.0366 (16)	0.0371 (15)	−0.0008 (12)	0.0003 (12)	−0.0153 (13)
C16	0.0455 (16)	0.0244 (14)	0.0444 (16)	0.0020 (11)	−0.0035 (12)	−0.0030 (12)
C17	0.0385 (14)	0.0330 (14)	0.0307 (14)	0.0009 (11)	−0.0038 (11)	−0.0015 (11)
C14	0.0423 (15)	0.0346 (15)	0.0274 (14)	−0.0040 (11)	0.0009 (11)	−0.0062 (11)

### Geometric parameters (Å, °)

**Table:**

Mn1—O1	2.1335 (15)	C10—H10	0.9300
Mn1—O1^i^	2.1335 (15)	C6—C7	1.378 (3)
Mn1—O1W^i^	2.1949 (16)	C6—C3	1.476 (3)
Mn1—O1W	2.1949 (16)	C7—C8	1.377 (4)
Mn1—N1	2.3038 (19)	C7—H7	0.9300
Mn1—N1^i^	2.3038 (19)	C8—H8	0.9300
F1—C13	1.361 (3)	C3—C4	1.385 (3)
F2—C15	1.345 (3)	C3—C2	1.389 (3)
F3—C16	1.354 (3)	C4—C5	1.374 (3)
O1—C11	1.266 (3)	C4—H4	0.9300
O1W—H1W	0.8445	C5—H5	0.9300
O1W—H2W	0.8541	C1—C2	1.369 (3)
O2—C11	1.242 (3)	C1—H1	0.9300
O3—C14	1.342 (3)	C2—H2	0.9300
O3—H3	0.8200	C11—C12	1.510 (3)
N1—C1	1.326 (3)	C12—C13	1.377 (3)
N1—C5	1.338 (3)	C12—C17	1.392 (3)
N2—C9	1.316 (3)	C13—C14	1.382 (3)
N2—C8	1.323 (3)	C15—C16	1.368 (3)
C9—C10	1.383 (4)	C15—C14	1.388 (3)
C9—H9	0.9300	C16—C17	1.363 (3)
C10—C6	1.374 (3)	C17—H17	0.9300
			
O1—Mn1—O1^i^	180.0	C7—C8—H8	118.2
O1—Mn1—O1W^i^	88.91 (6)	C4—C3—C2	115.2 (2)
O1^i^—Mn1—O1W^i^	91.09 (6)	C4—C3—C6	123.1 (2)
O1—Mn1—O1W	91.09 (6)	C2—C3—C6	121.7 (2)
O1^i^—Mn1—O1W	88.91 (6)	C5—C4—C3	121.1 (2)
O1W^i^—Mn1—O1W	180.00 (8)	C5—C4—H4	119.5
O1—Mn1—N1	89.39 (6)	C3—C4—H4	119.5
O1^i^—Mn1—N1	90.61 (6)	N1—C5—C4	123.2 (2)
O1W^i^—Mn1—N1	84.76 (7)	N1—C5—H5	118.4
O1W—Mn1—N1	95.24 (7)	C4—C5—H5	118.4
O1—Mn1—N1^i^	90.61 (6)	N1—C1—C2	124.6 (2)
O1^i^—Mn1—N1^i^	89.39 (6)	N1—C1—H1	117.7
O1W^i^—Mn1—N1^i^	95.24 (7)	C2—C1—H1	117.7
O1W—Mn1—N1^i^	84.76 (7)	C1—C2—C3	120.2 (2)
N1—Mn1—N1^i^	180.0	C1—C2—H2	119.9
C11—O1—Mn1	130.94 (15)	C3—C2—H2	119.9
Mn1—O1W—H1W	133.3	O2—C11—O1	125.9 (2)
Mn1—O1W—H2W	102.0	O2—C11—C12	118.6 (2)
H1W—O1W—H2W	104.9	O1—C11—C12	115.3 (2)
C14—O3—H3	109.5	C13—C12—C17	118.4 (2)
C1—N1—C5	115.8 (2)	C13—C12—C11	122.2 (2)
C1—N1—Mn1	122.51 (15)	C17—C12—C11	119.3 (2)
C5—N1—Mn1	121.30 (16)	F1—C13—C12	119.4 (2)
C9—N2—C8	116.3 (2)	F1—C13—C14	116.7 (2)
N2—C9—C10	123.8 (2)	C12—C13—C14	123.9 (2)
N2—C9—H9	118.1	F2—C15—C16	119.9 (2)
C10—C9—H9	118.1	F2—C15—C14	118.9 (2)
C6—C10—C9	120.1 (3)	C16—C15—C14	121.2 (2)
C6—C10—H10	119.9	F3—C16—C17	120.4 (2)
C9—C10—H10	119.9	F3—C16—C15	117.6 (2)
C10—C6—C7	115.8 (2)	C17—C16—C15	122.0 (2)
C10—C6—C3	122.7 (2)	C16—C17—C12	118.7 (2)
C7—C6—C3	121.5 (2)	C16—C17—H17	120.6
C8—C7—C6	120.4 (3)	C12—C17—H17	120.6
C8—C7—H7	119.8	O3—C14—C13	125.6 (2)
C6—C7—H7	119.8	O3—C14—C15	118.5 (2)
N2—C8—C7	123.6 (3)	C13—C14—C15	115.9 (2)
N2—C8—H8	118.2		
			
O1W^i^—Mn1—O1—C11	−155.2 (2)	Mn1—N1—C1—C2	−171.9 (2)
O1W—Mn1—O1—C11	24.8 (2)	N1—C1—C2—C3	−0.3 (4)
N1—Mn1—O1—C11	120.1 (2)	C4—C3—C2—C1	−0.6 (4)
N1^i^—Mn1—O1—C11	−59.9 (2)	C6—C3—C2—C1	179.3 (2)
O1—Mn1—N1—C1	−165.93 (19)	Mn1—O1—C11—O2	−10.1 (4)
O1^i^—Mn1—N1—C1	14.07 (19)	Mn1—O1—C11—C12	165.38 (14)
O1W^i^—Mn1—N1—C1	105.11 (19)	O2—C11—C12—C13	−128.6 (2)
O1W—Mn1—N1—C1	−74.89 (19)	O1—C11—C12—C13	55.6 (3)
O1—Mn1—N1—C5	21.15 (19)	O2—C11—C12—C17	55.3 (3)
O1^i^—Mn1—N1—C5	−158.85 (19)	O1—C11—C12—C17	−120.5 (2)
O1W^i^—Mn1—N1—C5	−67.81 (19)	C17—C12—C13—F1	−178.5 (2)
O1W—Mn1—N1—C5	112.19 (19)	C11—C12—C13—F1	5.4 (3)
C8—N2—C9—C10	−1.1 (5)	C17—C12—C13—C14	0.0 (4)
N2—C9—C10—C6	−0.6 (5)	C11—C12—C13—C14	−176.1 (2)
C9—C10—C6—C7	1.2 (4)	F2—C15—C16—F3	−0.3 (4)
C9—C10—C6—C3	−179.3 (2)	C14—C15—C16—F3	179.2 (2)
C10—C6—C7—C8	−0.2 (4)	F2—C15—C16—C17	179.4 (2)
C3—C6—C7—C8	−179.6 (3)	C14—C15—C16—C17	−1.0 (4)
C9—N2—C8—C7	2.3 (5)	F3—C16—C17—C12	−178.7 (2)
C6—C7—C8—N2	−1.7 (5)	C15—C16—C17—C12	1.5 (4)
C10—C6—C3—C4	14.1 (4)	C13—C12—C17—C16	−1.0 (3)
C7—C6—C3—C4	−166.5 (3)	C11—C12—C17—C16	175.2 (2)
C10—C6—C3—C2	−165.7 (2)	F1—C13—C14—O3	−2.3 (4)
C7—C6—C3—C2	13.6 (4)	C12—C13—C14—O3	179.2 (2)
C2—C3—C4—C5	0.5 (4)	F1—C13—C14—C15	179.1 (2)
C6—C3—C4—C5	−179.4 (2)	C12—C13—C14—C15	0.5 (4)
C1—N1—C5—C4	−1.5 (4)	F2—C15—C14—O3	0.7 (4)
Mn1—N1—C5—C4	171.9 (2)	C16—C15—C14—O3	−178.8 (2)
C3—C4—C5—N1	0.6 (4)	F2—C15—C14—C13	179.5 (2)
C5—N1—C1—C2	1.3 (4)	C16—C15—C14—C13	0.0 (4)

Symmetry codes: (i) −*x*, −*y*+1, −*z*+1.

### Hydrogen-bond geometry (Å, °)

**Table:**

*D*—H···*A*	*D*—H	H···*A*	*D*···*A*	*D*—H···*A*
O1W—H2W···O2	0.85	2.04	2.839 (2)	155.
O1W—H1W···O2^ii^	0.84	1.97	2.773 (2)	159.
O3—H3···N2^iii^	0.82	1.89	2.641 (3)	152.

Symmetry codes: (ii) −*x*+1, −*y*+1, −*z*+1; (iii) −*x*, −*y*+2, −*z*.
